# Supplementary material for: Pro- and Antioxidant Effect of Food Items and Matrices during Simulated In Vitro Digestion
Source: Foods. 2023 Apr 20;12(8):1719. doi: 10.3390/foods12081719 (PMC10137800; doi:10.3390/foods12081719)
Supplement: Supplementary file 1 [file foods-12-01719-s001.zip › foods-2289189-supplementary.pdf]

Supplementary Table S1: Food combinations for each digesta*.

**Condition(s)**	Food item 1	Food item 2	Quantity used per simulated digestion (Food item 1 + Food item 2)
1	Soda (Sprite)	Tomato Juice (Delhaize®)	2 mL + 2 mL
2	Soda	Orange Juice (Minute Maid)	2 mL + 2 mL
3	Soda	Vitamin C (Sigma-Aldrich)	2 mL + 2.5 mg
4	Soda	Vitamin E (Fisher Scientific)	2 mL + 1.5 mg
5	Soda	Coffee (Chaqwa®)**	2 mL + 2 ml
6	Soda	Curcumin (Apollo Scientific)	2 ml + 12.5 mg
7	Soda	-	2 mL
8	Sausage (Aoste)	Tomato Juice	2 g + 2 mL
9	Sausage	Orange Juice	2 g + 2mL
10	Sausage	Vitamin C	2 g + 2.5 mg
11	Sausage	Vitamin E	2 g + 1.5 mg
12	Sausage	Coffee	2 g + 2 mL
13	Sausage	Curcumin	2 g + 12.5 mg
14	Sausage	-	2 g
15	White Chocolate (Lindt)	Tomato Juice	2 g + 2 mL
16	White Chocolate	Orange Juice	2 g + 2 mL
17	White Chocolate	Vitamin C	2 g + 2.5 mg
18	White Chocolate	Vitamin E	2 g + 1.5 mg
19	White Chocolate	Coffee	2 g + 2 mL
20	White Chocolate	Curcumin	2 g + 12.5 mg
21	White Chocolate	-	2 g
22	Tomato Juice	-	2 mL
23	Orange Juice	-	2 mL
24	Vitamin C	-	2.5 mg
25	Vitamin E	-	1.5 mg
26	Coffee	-	2 mL
27	Curcumin	-	12.5 mg
28	Coffee	Vitamin C	2 mL + 2.5 mg
29	Coffee	Orange Juice	2 mL + 2 mL
30	Vitamin E	Vitamin C	1.5 mg + 2.5 mg
31	Sausage	White Chocolate	2 g + 2 g
32	Soda	White Chocolate	2 mL + 2 g
33	Sausage	Soda	2 g + 2 mL

* More details on the composition of food items are given in the Materials and Methods section: 2.2. Food items and matrices (Table 1). All food items were purchased from a local supermarket (Delhaize®, Strassen, Luxembourg).

** Coffee machine model: Stand-Alone Automatique, Anderlecht, Belgium.

Supplementary Table S2: Stock solutions used for preparing gastric (SGF) and intestinal fluids (SIF) for digestion.

Solute	Stock Solution		SGF (108 mL)		SIF (216 mL)	
	Concentration		Concentration	Vol. Stock	Concentration	Vol. Stock
	mol/L	g/100 mL	mmol/L	mL	mmol/L	mL
**KCl**	0.50	3.73	6.9	1.87	6.8	3.68
**KH_2PO_4**	0.50	6.80	0.9	0.24	0.8	0.43
**$NaHCO_3$**	1.00	21.00	25	3.38	85	22.98
**NaCl**	2.00	11.69	47.2	3.19	38.4	5.19
**$MgCl_2(H_2O)_6$**	0.15	3.05	0.1	0.09	0.33	0.59
**$(NH_4)_2CO_3$**	0.50	4.80	0.5	0.14	0	0
**$CaCl_2(H_2O)_2$**	0.03	0.441	0.075	32.5	0.3	260

Supplementary Table S3: Standard preparation for FRAP assay.

Standard	Iron (II) chloride concentration (mg/L)	Dilution	Iron (II) chloride stock solution (μL)	Distilled water (μL)
**Blank**	0	0	0	1000
**Standard 1**	6.34	1:400	2.5	997.5
**Standard 2**	12.68	1:200	5	995
**Standard 3**	19.02	1:333,33	7.5	992.5
**Standard 4**	25.36	1:100	10	990
**Standard 5**	38.04	1:66,66	15	985
**Standard 6**	50.72	1:50	20	980
**Standard 7**	76.09	1:33,33	30	970
**Standard 8**	101.44	1:25	40	960
**Standard 9**	126.8	1:20	50	950

Supplementary Table S4: Ascorbic acid standards employed for the ABTS assay.

Standard	Concentration (mg/mL)	Dilution	Ascorbic acid stock solution (μL)	Distilled water (μL)
**Blank**	0	0	0	1000
**Standard 1**	0.05	1:200	5	995
**Standard 2**	0.1	1:100	10	990
**Standard 3**	0.2	1:50	20	980
**Standard 4**	0.3	1:33,33	30	970
**Standard 5**	0.4	1:25	40	960
**Standard 6**	0.5	1:20	50	950

Supplementary Table S5: MDA (malondialdehyde) calibration curve dilutions.

Sample	MDA 25 μM (μL)	Distilled water (μL)	MDA concentration (μM)
**A**	0	500	0
**B**	2.5	498	0.125
**C**	5	495	0.25
**D**	10	490	0.5
**E**	20	480	1
**F**	50	450	2.5
**G**	100	400	5
**H**	200	300	10
**I**	400	100	20
